# A20 deficiency in hematopoietic stem cells causes lymphopenia and myeloproliferation due to elevated Interferon-γ signals

**DOI:** 10.1038/s41598-019-49038-8

**Published:** 2019-09-02

**Authors:** Masahiro Marshall Nakagawa, Chozha Vendan Rathinam

**Affiliations:** 1 Department of Genetics and Development, Columbia University Medical Center, 701W 168th street, New York, NY 10032 USA; 2 Institute of Human Virology, University of Maryland, School of Medicine, Baltimore, MD 21201 USA; 3 Center for Stem Cell & Regenerative Medicine, University of Maryland, School of Medicine, Baltimore, MD 21201 USA; 4 Marlene & Stewart Greenebaum Comprehensive Cancer Center, 725W Lombard Street, University of Maryland, School of Medicine, Baltimore, MD 21201 USA

**Keywords:** Haematopoietic stem cells, Stem-cell differentiation

## Abstract

Inflammation and inflammatory cytokines have been shown to exert both positive and negative effects on hematopoietic stem cells (HSCs) and hematopoiesis. While the significance of inflammation driven hematopoiesis has begun to unfold, molecular players that regulate this phenomenon remain largely unknown. In the present study, we identified A20 as a critical regulator of inflammation controlled hematopoietic cell fate decisions of HSCs. A20 deficiency in HSCs leads to increased differentiation of myeloid cells and myeloproliferation. Analysis of erythroid lineage cells of A20 deficient mice indicated a striking reduction of erythrocytes in the bone marrow (BM), but elevated numbers in the spleen. Loss of A20 in HSCs causes a severe blockade of B cell differentiation in the BM and absence of peripheral B cells in the spleen, liver and blood. T cell differentiation studies revealed a reduction of both T cell progenitors and differentiated T cells in the thymus and altered T cell numbers in the spleens of A20 mutant mice. Analysis of lineage committed progenitors of the myeloid, erythroid and lymphoid lineages specified an altered composition in the A20 deficient BM. Genetic studies identified that specific loss of A20 in the myeloid lineage cells results in myeloproliferation. Bone marrow transplantation studies and mixed bone marrow chimera studies suggested an involvement of inflammatory cytokines, particularly interferon (IFN)- γ, in the onset of myeloproliferation and lymphopenia of A20 deficient mice. Finally, ablation of IFNγ signals in A20 deficient mice rescued the hematopoietic defects. In essence, these studies highlight a previously unknown role for A20 in the restriction of inflammation driven pathologic hematopoiesis. We believe that our studies based on A20 mutant mice will be helpful in understanding the pathophysiology and in the treatment of patients with A20 (*TNFAIP3*) mutations.

## Introduction

Hematopoiesis is a process through which blood cells are constantly generated and replenished in the body. A tight regulation on proliferation and differentiation of HSCs into lineage committed progenitors is vital for maintaining a balance between myeloid and lymphoid lineage cells in the blood. Indeed defective regulation of HSC proliferation and/or differentiation can lead to detrimental consequences, including myelodysplasia, lymphopenia, immunodeficiencies, anemia, myeloproliferation, leukemia and lymphoma^1,2^. During myelopoiesis, HSCs differentiate into Multipotent Progenitors (MPPs), which further differentiate into common myeloid progenitors (CMPs). These CMPs give rise to either granulocyte monocyte progenitors (GMPs), that differentiate into granulocytes, monocyte/macrophages and dendritic cells, or Megakaryocyte erythrocyte progenitors (MEPs) that differentiate into either erythrocytes or megakaryocytes^3^. Differentiation of majority of these myeloid lineage cells occurs in the BM. On the other hand, during lymphopoiesis, MPPs differentiate into common lymphoid progenitors (CLPs), which either differentiate into B cell and natural killer (NK) cell progenitors in the BM or migrate to the thymus to generate early thymic progenitors (ETPs), which give rise to both cytotoxic and helper T cells, and NKT cells through a series of differentiation steps^4^.

A constellation of cell intrinsic and extrinsic factors regulate these specific stages of differentiation from HSCs. To date various cell intrinsic factors, including transcription factors, cell cycle regulators, microRNAs and signal transducers, and extrinsic factors, such as cytokines, chemokines, and signals from the BM “niche”, have been shown to control hematopoiesis^5,6^. We and others have shown the significance of post-translational modifications of proteins, especially ubiquitylation, in hematopoiesis. Loss functions mediated by the E3 ubiquitin ligase c-Cbl results in compromised HSC functions^7^, age related myeloproliferation and lymphopenia^8^, and the onset of acute myeloid leukemia^9^. Similarly, deficiency of another HECT type E3 ubiquitin ligase-Itch causes abnormal hematopoiesis^10^. More recently, we have shown that a deficiency of the ubiquitin editing enzyme-A20 causes increased NF-κB activation that results in premature death, due to compromised HSC pool and functions^11^.

A20 (Tnfaip3) is a broadly expressed cytoplasmic protein that was originally identified as an inhibitor of TNF-induced NF-κB activity^12,13^. A20 is induced by NF-κB signals and is regulated at both transcriptional and post-transcriptional levels^14^. It plays a critical role in determining the duration and intensity of signaling by many components of the NF-κB pathway. A20 has been shown to interact with a variety of signaling molecules including TRAF1, TRAF2, TRAF6 and NEMO, therefore believed to regulate many other inflammatory pathways^15^. Mice deficient for A20 exhibit hypersensitivity to TNF and premature death due to severe inflammation and cachexia^16^. Deletion of A20 in specific cells of the immune system, including B cells^17^, T cells^18^, dendritic cells^19^, and myeloid cells^20^ resulted in a variety of multi-organ inflammation and immune pathologies^14^. Consistently, Mx1-Cre or ERT2-Cre mediated ablation of A20 in mice resulted in increased myeloid differentiation and rapid B cell apoptosis^21^. While all these studies have highlighted the importance of A20 in the functions of specific immune cell types, it remained unclear if A20 has any roles on hematopoietic differentiation, especially at the earlier stages of myeloid, erythroid and lymphoid lineages. Even though we have recently shown that deficiency of A20 leads to loss of quiescence and maintenance of HSCs due to exaggerated IFNγ signals^11^, it was unknown if exaggerated IFNγ mediated signals are responsible for the onset of myeloproliferation and lymphopenia caused by A20 deficiency. In the present study we provide evidence that A20 deficiency in myeloid lineage cells is sufficient to cause myeloproliferation and reduction of peripheral B cells. In addition, our data demonstrate that ablating IFNγ signals is sufficient to rescue the pathologic hematopoiesis caused by A20 deficiency.

## Materials and Methods

### Mice

A20^HemKO^ (Tnfaip3^flox/flox^ mice crossed with Vav-iCre mice) mice were previously described^11^. VAV^Cre/+^ mice, CD19^cre/+^ mice, LysM^cre/+^ mice and IFN-γ^−/−^ mice were purchased from the Jackson laboratory. CD45.1 congenic animals were purchased from the National Cancer Institute. Mice were maintained under specific pathogen–free conditions and used according to the protocols approved by the Institutional Animal Care and Use Committees (IACUC) of Columbia University Medical Center and University of Maryland School of Medicine.

### Cell preparation

A20^Hem-KO^ mice were analyzed at 14 days after birth (P14), unless otherwise specified. Bone marrow cells were isolated from the tibias and femurs by inserting a 23-gauge needle/1 mL syringe to the bone cavities and flushed with PBS 2%FCS until the bones become pale. Single cell suspensions were made through rigorous pipetting. Red blood cells were lysed with Ammonium chloride (Stem Cell Technology) and subsequently filtered using a 70 nM nylon mesh. Bone marrow cells were then counted with a hemacytometer and trypan blue (Amresco) negative cells were counted as live cells. Peripheral blood cells were lysed with ice cold H_2_0 and followed by ice cold 2.7% Nacl. Cells were filtered using a 70 nM nylon mesh.

### Flow cytometry

Cells were analyzed by flow cytometry with FACSForettsa or LSR II (BD) and FACSDiva software (BD Biosciences) or FlowJo software (Tree Star). The following monoclonal antibodies were used: anti-B220 (RA3–6B2), anti- CD19 (1D3), anti- CD3ε (145-2C11), anti-CD4 (GK1.5), anti-CD8 (53-6.7), anti-CD11b (M1/70), anti-CD34 (RAM34), anti-CD45.1 (A20), anti-CD45.2 (104), anti-CD48 (HM48-1), anti-CD117 (2B8), anti-Flt3 (A2F10.1), anti-Gr-1 (RB6-8C5), anti-Sca-1 (D7), anti-CD71 (C2), anti-CD44 (IM7), anti-CD62L (MEL-14) and anti-Ter119 (TER119); from BD Biosciences; anti-CD25 (PC61) and anti-CD150 (TC15- 12F12.2) from Biolegend; anti-CD16/32 (93), anti-Ly6D (49-H4), anti-CD11C (N418) anti-NK1.1 (PK136)anti-CD41 (MWReg30), anti-CD105 (MJ7/18) and anti-CD127 (A7R34) from eBioscience. Cells incubated with biotinylated monoclonal antibodies were incubated with fluorochrome-conjugated streptavidin–Peridinin Chlorophyllprotein–Cyanine 5.5 (551419; BD Biosciences) or Streptavidin-Allophycocyanin-Cy7 (554063; BD Biosciences). In all the FACS plots, indicated are the relative frequencies (%) of the gated fraction.

### Intracytoplasmic Staining

Surface stained splenocytes from control and A20^Hem-KO^ mice were fixed and permeabilized using Fixation/Permeabilization solution kit (BD biosciences). Cells were treated with IFN_γ_-PE (XMG1.2) antibodies to detect intracellular IFN_γ_ levels in various immune subsets.

### Apoptosis assays

Apoptotic cells were detected with the Annexin V PE Apoptosis Detection kit or Annexin V FITC Apoptosis Detection kit (BD) according to the manufacturer’s instructions.

### Bone marrow transplantation studies

1 × 10^6^ of bone marrow cells were injected into lethally irradiated (10 Gy) congenic (CD45.1^+^) recipient mice. For competitive-repopulation experiments, 1 × 10^6^ of bone marrow cells from either A20 control or A20^Hem-KO^ mice were mixed with 2 × 10^5^ of wild type (WT; CD45.1^+^) bone marrow cells (to obtain a ratio of 5:1, respectively) and were injected into lethally irradiated congenic WT (CD45.1^+^) recipient mice.

### Colony forming unit (CFU) assays

Either Total BM (5 × 10^4^ cells/1 mL in 3 cm dish) or purified hematopoietic progenitors (100 cells/1 ml in 3 cm dish) were mixed with methylcellulose medium with recombinant cytokines for mouse cells (M3434; Stemcell Technologies). Cells were plated in 3 independent dishes, cultured and myeloid colonies were scored after 10 days as described in the manual (Stemcell Technologies).

### RNA extraction real-time PCR

Total RNA was isolated with an RNeasy Mini kit (Qiagen), then cDNA was synthesized with oligo(dT) primer and Maxima reverse transcriptase (Thermo Scientific). Real-time PCR was performed using gene specific primers (Supplemental Table 1) in duplicates with a CFX-connect RealTime PCR system (BioRad) and SsoAdvanced SYBR Green Supermix according to the manufacturer’s instructions (BioRad). Relative expression was normalized to the expression levels of the internal control-HPRT.

### Statistical analyses

We used the unpaired Student’s t test for all the experiments but survival analysis, and logrank test for survival analysis. Differences with P < 0.05 were considered statistically significant and denoted as *P < 0.05, **P < 0.01, ***P < 0.001.

## Results

### Lack of A20 in hematopoietic cells leads to myeloproliferation

In an attempt to identify the functions of the ubiquitin editing enzyme A20 in HSCs, we previously generated mice that specifically lack A20 in HSCs (and in all their progeny), by crossing A20 ^F/F^ mice with Vav^cre/+^ transgenic mice to generate A20^F/F^ Vav^cre/+^ (A20^Hem-KO^)^11,22^. Our analysis of A20^Hem-KO^ mice revealed that A20 deficiency leads to perinatal lethality and loss of HSPC pool and functions.

**Figure 1:**
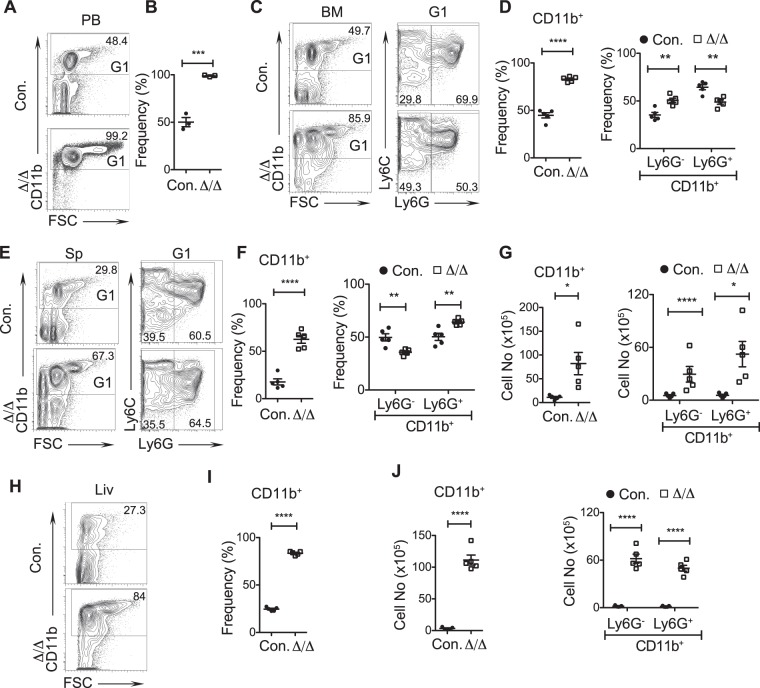
A20 deficiency in hematopoietic cells leads to myeloproliferation. (**A**,**B**) FACS plots (**A**) and frequencies (**B**) of myeloid cells from the peripheral blood (PB) of 14 days old A20^Hem-KO^ and control mice (n = 3). (**C**,**D**) FACS plots (**C**) and frequencies (**D**) of myeloid cells from the bone marrow (BM) of 14 days old A20^Hem-KO^ and control mice (n = 5). (**E**–**G**) FACS plots (**E**), frequencies (**F**) and absolute numbers (**G**) of myeloid cells from the spleen (Sp) of 14 days old A20^Hem-KO^ and control mice (n = 5). (**H**–**J**) FACS plots (**H**), frequencies (**I**) and absolute numbers (**J**) of myeloid cells from the liver (Liv) of 14 days old A20^Hem-KO^ and control mice (n = 5). All data represent mean ± SEM. Two-tailed student’s t tests were used to assess statistical significance (*P < 0.05, **P < 0.01, ***P < 0.001).

While our previous studies^11^ specified the significance of A20 in HSC quiescence and functions, importance of A20 in hematopoietic differentiation remains to be explored. To this end, we performed immunophenotyping studies to study myeloid, erythroid and lymphoid differentiation in the absence of A20. Our analysis of peripheral blood indicated ~48% of CD11b^+^ cells in control animals, whereas ~99% of CD11b^+^ cells were noticed in A20^Hem-KO^ mice (Fig. 1A,B). To identify if the increased propensity of myeloid cells in the blood was due to increased differentiation or migration, we enumerated their frequencies in the bone marrow (BM). While control BM had ~50% of CD11b^+^ cells, BM of A20^Hem-KO^ mice had >85% of CD11b^+^ cells (Fig. 1C,D). Further analysis of myeloid cells indicated a relative increase (~30% in control and ~50% in A20^Hem-KO^) of Ly6G^−^CD11b^+^ subset and a decrease (~70% in control and ~50% in A20^Hem-KO^) of Ly6G^+^CD11b^+^ granulocytes in the BM of A20^Hem-KO^ mice (Fig. 1C,D). Consistent with an increase of myeloid cells in the BM, analysis of spleen revealed a striking relative increase (~29% in control and ~67% in A20^Hem-KO^) of CD11b^+^ cells in the spleen of A20^Hem-KO^ mice (Fig. 1E,F). Further characterization of the myeloid compartment suggested a relative decrease of Ly6G^−^CD11b^+^ subset and an increase of Ly6G^+^CD11b^+^ granulocytes in the spleen of A20^Hem-KO^ mice (Fig. 1E,F). We have previously reported that A20 deficiency leads to splenomegaly and that A20 deficient spleens have 5–10 fold more total cells than control spleens^11^. To assess if the absolute numbers of myeloid cells were also increased in the spleens of A20^Hem-KO^ mice, we enumerated the numbers of total CD11b^+^, Ly6G^−^CD11b^+^ and Ly6G^+^CD11b^+^ subsets in the spleen and data indicated increased numbers of all these 3 subsets (Fig. 1G).

Finally, we assessed the frequencies of CD11b^+^ cells in the liver, as A20 deficiency leads to hepatomegaly and an infiltration of leukocytes in the liver^11^. As expected, CD11b^+^ cells were remarkably increased (~27% in control and ~84% in A20^Hem-KO^), both in relative and absolute numbers in the livers of A20^Hem-KO^ mice (Fig. 1H–J). Further discrimination of CD11b^+^ subset indicated an increase in absolute numbers of both Ly6G^−^CD11b^+^ and Ly6G^+^CD11b^+^ fractions in the liver of A20^Hem-KO^ mice (Fig. 1J). Taken together, these data provide evidence that A20 deficiency leads to increased differentiation of myeloid cells and myeloproliferation.

### Defective erythroid differentiation in the absence of A20

**Figure 2:**
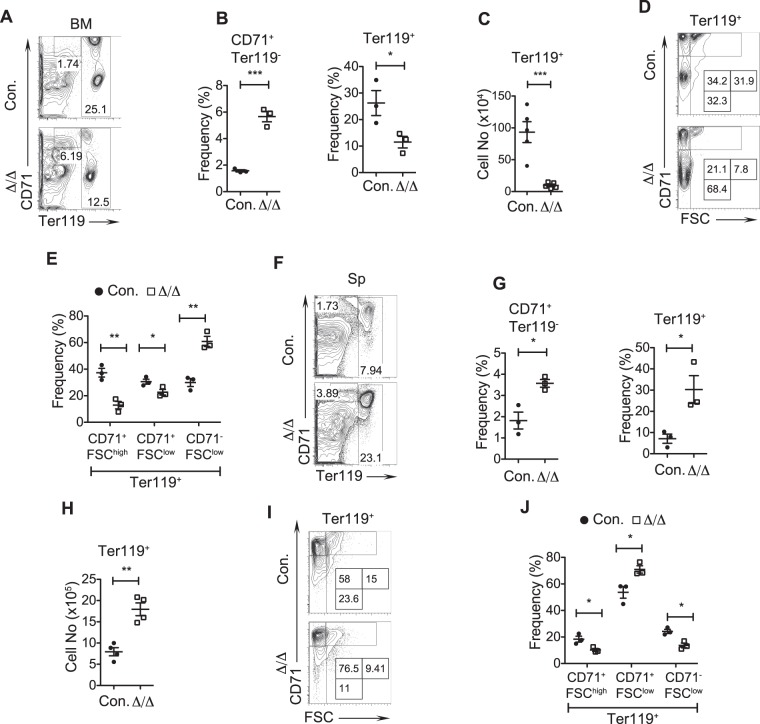
Erythroid differentiation is perturbed in A20 deficient mice. (**A**–**C**) FACS plots (**A**), frequencies (**B**) and absolute numbers (**C**) of erythroid cells from the bone marrow (BM) of 14 days old A20^Hem-KO^ and control mice (n = 3–5). (**D**,**E**) FACS plots (**D**) and frequencies (**E**) exhibiting differentiation of erythroid cells (EryA to EryC) in the BM of 14 days old A20^Hem-KO^ and control mice (n = 3). (**F**–**H**) FACS plots (**F**), frequencies (**G**) and absolute numbers (**H**) of erythroid cells from the spleen of 14 days old A20^Hem-KO^ and control mice (n = 3–4). (**I**,**J**) FACS plots (**I**) and frequencies (**J**) exhibiting differentiation of erythroid cells (EryA to EryC) in the spleen (Sp) of 14 days old A20^Hem-KO^ and control mice (n = 3). All data represent mean ± SEM. Two-tailed student’s t tests were used to assess statistical significance (*P < 0.05, **P < 0.01, ***P < 0.001).

To assess differentiation of HSCs into the erythroid lineage, we performed detailed immunophenotyping studies using CD71 and TER119 antibodies. Expression levels of these surface antigens identify erythroid lineage cells at distinct stages of development^23^. Our analysis of A20^Hem-KO^ BM revealed an accumulation of CD71^hi^TER119^−^ cells (Fig. 2A,B), an aberrant erythroid lineage subset that was previously identified as the r2-r4a erythroblast cells that fail to express TER119^24^. However, the relative and absolute frequencies of erythroid lineage committed (TER119^high^) cells were reduced in the BM of A20^Hem-KO^ mice (Fig. 2A–C). To further identify distinct stages of erythroblasts (EBs) we adopted a previously described staining scheme that resolves three erythroblast subpopulations previously labeled as EryA (Ter119^high^CD71^high^FSC^high^), EryB (Ter119^high^CD71^high^FSC^low^) and EryC (Ter119^high^CD71^low^FSC^low^)^23^, based on expression levels of Ter119 and CD71, and in combination with forward scatter (FSc) parameter^23^. Accordingly, EryA corresponds to baso-EBs; EryB corresponds to late baso-EBs and polychromatic-EBs; EryC are orth-EBs and reticulocytes. Our analysis of BM from A20^Hem-KO^ mice indicated while EryA and EryB subsets were reduced (Fig. 2D,E), the frequencies of EryC were remarkably increased (Fig. 2D,E). Next, we analyzed the splenic erythropoiesis of A20^Hem-KO^ mice. Consistent with the BM, the frequencies of CD71^hi^TER119^−^ fraction were increased in the spleen (Fig. 2F,G). However, in sharp contrast to the BM, both relative and absolute numbers of total TER119^+^ cells were increased in the spleens of A20^Hem-KO^ mice (Fig. 2G,H). Further detailed analysis of TER119^high^ cells revealed a decrease in EryA and EryC subsets, while an increase of EryB subset was observed in the spleens of A20^Hem-KO^ mice (Fig. 2I,J). These data are in support of our previous observation that A20^Hem-KO^ mice develop anemia^11^. Overall, these data suggest that A20 deficiency displaces erythropoiesis from BM to spleen.

### A20 deficiency results in perturbed B cell differentiation

**Figure 3:**
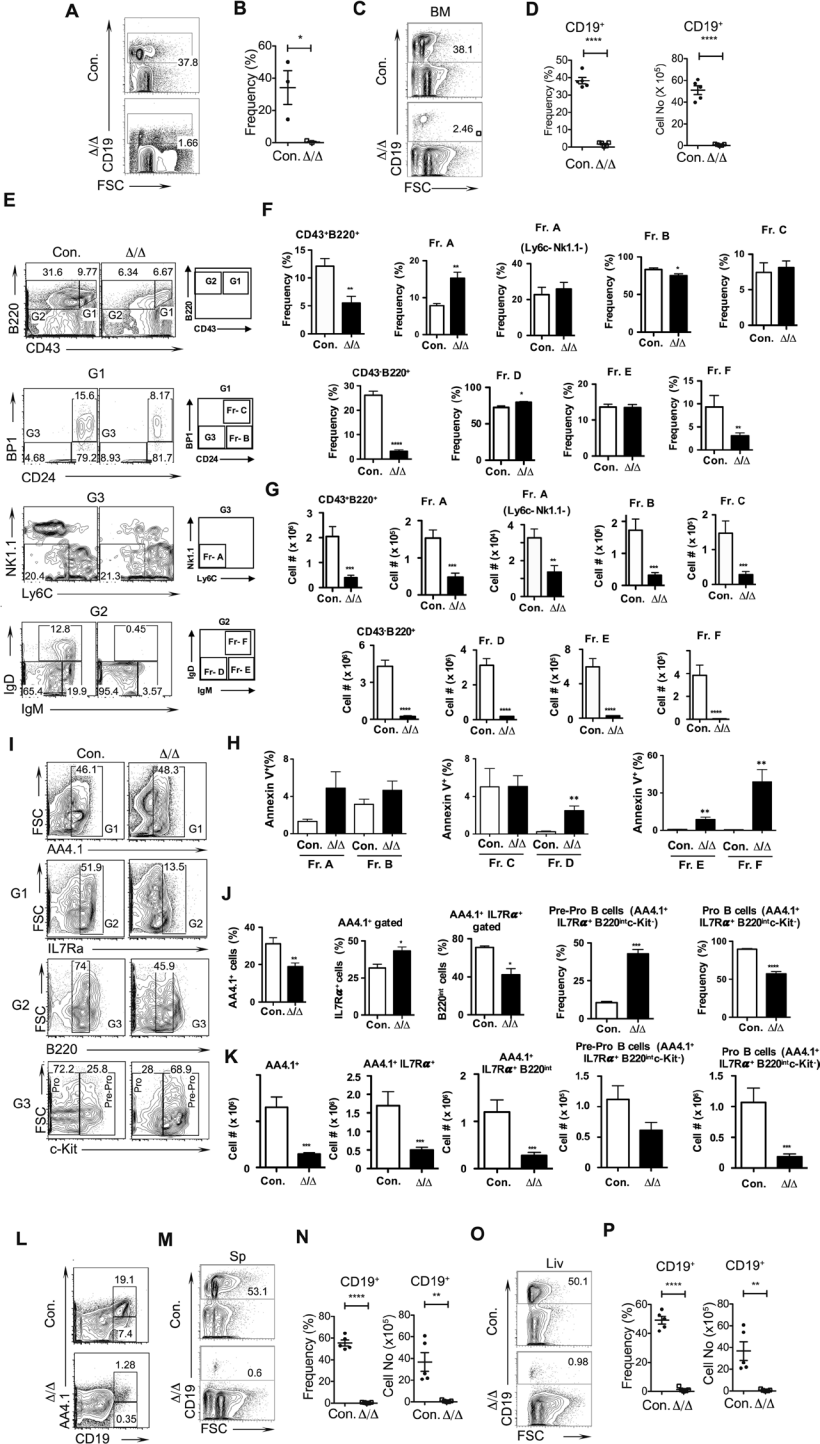
Loss of A20 causes impaired B cell differentiation. (**A**,**B**) FACS plots (**A**) and frequencies (**B**) of B cells from the peripheral blood of 14 days old A20^Hem-KO^ and control mice (n = 3). (**C**,**D**) FACS plots (**C**), frequencies and absolute numbers (**D**) of B cells from the bone marrow (BM) of 14 days old A20^Hem-KO^ and control mice (n = 5). (**E**–**G**) Hardy’s Fraction analysis of B cell development. FACS plots (**E**), frequencies (**F**) and absolute numbers (**G**) of B cell subsets from the BM of 14 days old A20^Hem-KO^ (n = 10) and control (n = 5) mice. (**H**) Frequencies of Annexin V^+^ cells in Hardy’s Fraction subsets of BM from 14 days old A20^Hem-KO^ (n = 4–8) and control (n = 5–7) mice. (**I**–**K**) B cell progenitor analysis. FACS plots (**I**), frequencies (**J**) and absolute numbers (**K**) of B cell subsets from the BM of 14 days old A20^Hem-KO^ (n = 10) and control (n = 5) mice. (**L**) FACS plots of B cell subsets from the BM of 14 days old A20^Hem-KO^ (n = 15) and control (n = 5) mice. (**M**,**N**) FACS plots (**M**), frequencies and absolute numbers (**N**) of B cells from the spleen (Sp) of 14 days old A20^Hem-KO^ and control mice (n = 5). (**O**,**P**) FACS plots (**O**), frequencies and absolute numbers (**P**) of B cells from the liver (Liv) of 14 days old A20^Hem-KO^ and control mice (n = 5). All data represent mean ± SEM. Two-tailed student’s t tests were used to assess statistical significance (*P < 0.05, **P < 0.01, ***P < 0.001).

To investigate the abilities of A20 deficient HSCs to differentiate into B cells, first we analyzed peripheral blood of A20^Hem-KO^ mice. Flow cytometric analysis suggested a striking reduction (37% in control and 1.6% in KO) of CD19^+^ B cells in the peripheral blood of A20^Hem-KO^ mice (Fig. 3A,B). Second, we studied the B cells of the BM, as B cell differentiation occurs primarily in the BM. Consistent with decreased CD19^+^ cells in the blood, analysis of BM revealed a remarkable reduction (38% in control and 2% in KO), in both relative and absolute numbers, of CD19^+^ cells in the BM of A20^Hem-KO^ mice (Fig. 3C,D). To identify the precise stage in which B cell development is perturbed in A20^Hem-KO^ mice, we performed Hardy’s Fraction analysis^25,26^. Accordingly, B cell development in the BM proceeds through at least 5 distinct stages that can be identified based on their immunophenotype; Fraction A (pre-proB cells; B220^+^CD43^+^CD24^−^BP1^−^Ly6C^−^NK1.1^−^); Fraction B (proB & PreB-I cells; B220^+^CD43^+^CD24^+^BP1^−^); Fraction C (Large PreB-II cells; B220^+^CD43^+^CD24^+^BP1^+^); Fraction D (Small PreB-II cells; B220^+^CD43^−^IgM^−^IgD^−^); Fraction E (Immature B cells; B220^+^CD43^−^IgM^+^IgD^−^); and Fraction F (Mature B cells; B220^+^CD43^−^IgM^+^IgD^+^). Our analysis of A20^Hem-KO^ mice revealed normal frequencies of Fraction A, Fraction C and Fraction E, modestly reduced frequencies of Fraction B and increased frequencies of Fraction D, and a strikingly reduced frequencies of Fraction F (Fig. 3E,F). On the other hand, enumeration of absolute numbers of these subsets indicated a significant reduction of all subsets (Fraction A-F), Fractions E &F were the most severely affected subsets, in the BM of A20^Hem-KO^ mice (Fig. 3G). To test if reduced B cell differentiation in the BM of A20 deficient mice can be explained by increased cell death^21,27^ of B cell committed progenitors, we performed apoptosis studies. Data indicated that the frequencies of Annexin V^+^ cells were comparable in early stages of B cell differentiation (Fractions A-C), however, the frequencies of apoptotic cells were remarkably increased at latter stages of differentiation (Fractions D-F) in the BM of A20^Hem-KO^ mice (Fig. 3H). These data are consistent with our previous findings^11^ that A20 deficiency does not induce apoptosis of hematopoietic progenitors. To further characterize these progenitor B cells, we followed an independent immunophenotyping strategy to identify Pre-Pro B (AA4.1^+^IL-7Ra^+^B220^int^c-Kit^+^) and Pro B (AA4.1^+^IL-7Ra^+^B220^int^c-Kit^−^) cells of the BM. Following this scheme of characterization, we identified that the relative frequencies of AA4.1^+^IL-7Ra^+^ cells were increased and the absolute numbers were decreased, and the relative frequencies and absolute numbers of AA4.1^+^IL-7Ra^+^B220^int^ were reduced in the BM of A20^Hem-KO^ mice (Fig. 3I–K). Further characterization of AA4.1^+^IL-7Ra^+^B220^int^ cells into Pre-ProB cells suggested an increase in relative numbers and a decrease in absolute numbers (although statistically not significant), and a decrease in both relative and absolute numbers of ProB cells in the BM of A20^Hem-KO^ mice (Fig. 3I–K). Consistently, characterization of B cells based on CD19 and AA4.1 identified a severe reduction of both AA4.1^+^CD19^+^ and AA4.1^−^CD19^+^ subsets in the BM of A20^Hem-KO^ mice (Fig. 3L). Third, we studied the frequencies of B cells in the spleen and our data indicated a complete loss (53% in control and 0.6% in KO) of B cells, both in relative and absolute numbers, in the spleen of A20^Hem-KO^ mice (Fig. 3M,N). Finally, we looked at the frequencies of B cells in the liver. As observed in the BM and spleen, CD19^+^ B cells were almost absent (50% in control and 0.9% in KO) in the livers of A20^Hem-KO^ mice (Fig. 3O,P). These data suggest that B cell development in the BM and maintenance of B cells in the peripheral organs (including spleen, liver and blood) is almost abolished in the absence of A20.

### Absence of A20 diminishes T cell development

**Figure 4:**
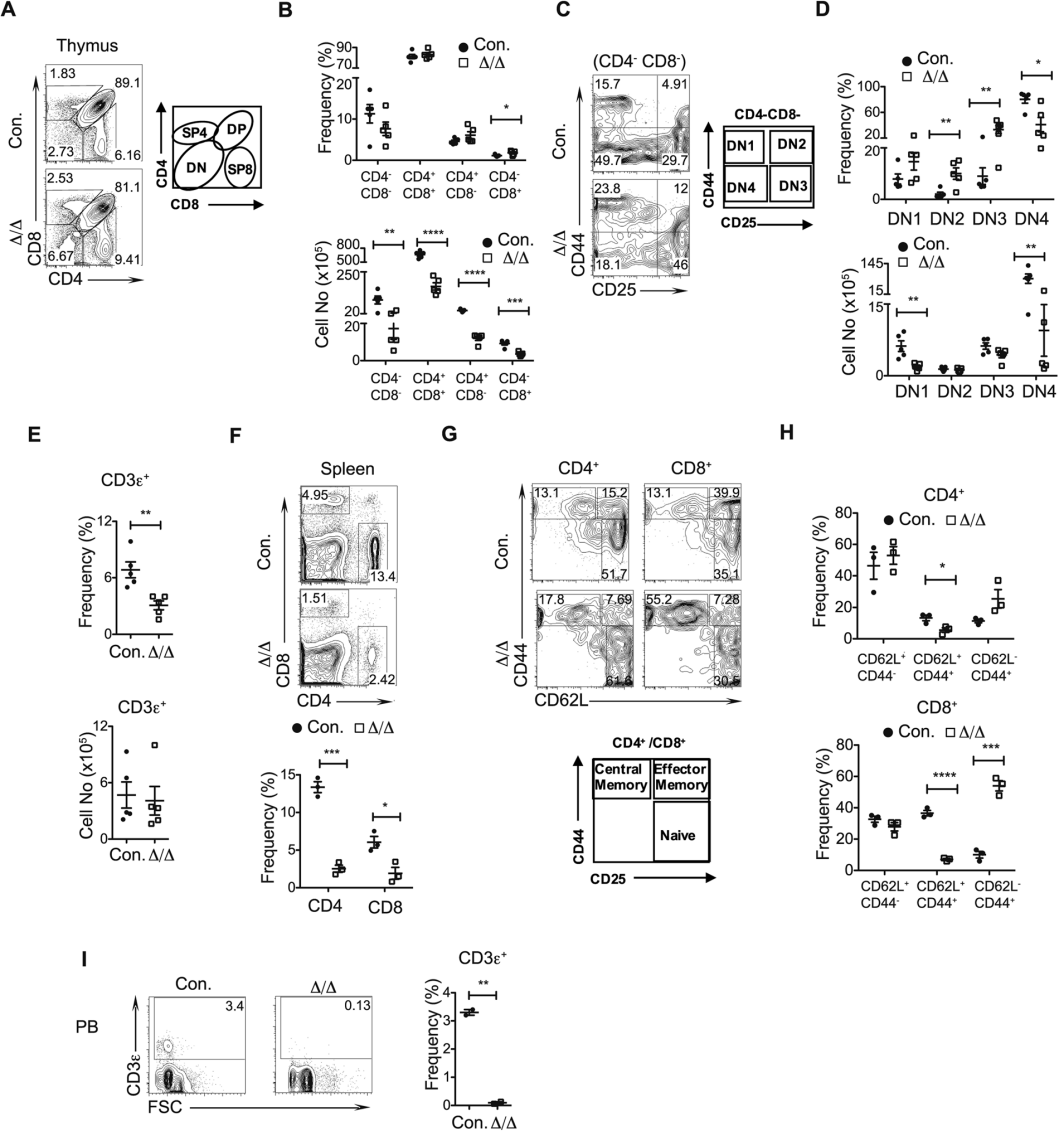
Deficiency of A20 results in impaired T cell development. (**A**–**D**) FACS plots (**A**,**C**), frequencies and absolute numbers (**B**,**D**) exhibiting differentiation of T cells from the thymus of 14 days old A20^Hem-KO^ and control mice (n = 5). (**A**,**B**) Double negative cells to single positive T cells; (**C**,**D**) DN1 to DN4 cells. (**E**) Frequencies of T cells from the spleen of 14 days old A20^Hem-KO^ and control mice (n = 5). (**F**) FACS plots and frequencies of CD4^+^ T cells and CD8^+^ T cells from the spleen of 14 days old A20^Hem-KO^ and control mice (n = 3). (**G**,**H**) FACS plots (**G**), frequencies and absolute numbers (**H**) of naïve, effector and memory T cells from the spleen of 14 days old A20^Hem-KO^ and control mice (n = 3). (**I**) FACS plots and frequencies of T cells from the peripheral blood (PB) of 14 days old A20^Hem-KO^ and control mice (n = 2). All data represent mean ± SEM. Two-tailed student’s t tests were used to assess statistical significance (*P < 0.05, **P < 0.01, ***P < 0.001).

To study the role of A20 in T cell differentiation, we analyzed thymic subsets of A20^Hem-KO^ mice. Even though our previous studies documented a reduction of ~90% total cellularity of the thymus^11^, immunophenotyping studies indicated an intact T cell development with normal relative frequencies of all 4 major subsets^28,29^; including CD4^−^CD8^−^ (Double Negative; DN), CD4^+^CD8^+^ (Double Positive; DP), CD4^+^ CD8^−^ (Single Positive 4; SP4) and CD4^−^ CD8^+^ (Single Positive 8; SP8) subsets, even though a modest increase of SP8 subset was noticed in A20^Hem-KO^ mice (Fig. 4A,B). Enumeration of absolute cell counts demonstrated a striking decrease of DN, DP, SP4 and SP8 subsets in A20^Hem-KO^ thymus (Fig. 4B). T cell development in the thymus proceeds from DN stage to DP stage and further to either SP4 or SP8, to differentiate into either helper T cells or cytotoxic T cells, respectively^28,29^. The DN subset of the thymus is further differentiated into 4 subsets based on the surface expression of CD44 and CD25; including CD44^+^CD25^−^ (DN1), CD44^+^CD25^+^ (DN2), CD44^−^CD25^+^ (DN3), and CD44^−^CD25^−^ (DN4), of which T cell differentiation proceeds from DN1 to DN2 to DN3 and to DN4^28,29^. Analysis of relative numbers of DN subsets indicated normal frequencies of DN1, and augmented frequencies of DN2, DN3 and DN4 subsets in the thymus of A20^Hem-KO^ mice (Fig. 4C,D). Absolute cell counts indicated decreased numbers of DN1 and DN4 subsets, but normal numbers of DN2 and DN3 subsets in the thymus of A20^Hem-KO^ mice (Fig. 4D). Next, we analyzed the frequencies of T cells in the spleen. While the overall relative numbers of total T (CD3ε^+^) cells were reduced, the absolute numbers of CD3ε^+^ were normal in the spleens of A20^Hem-KO^ mice (Fig. 4E). However, analysis of CD4^+^ and CD8^+^ T cell subsets indicated a reduction of both CD4^+^ and CD8^+^ subsets in the spleens of A20^Hem-KO^ mice (Fig. 4F). Furthermore, discrimination of either CD4^+^ or CD8^+^ T cells into functionally distinct subsets; naïve (CD44^low^CD62L^+^), effector (CD44^+^CD62L^+^) and memory (CD44^+^CD62L^−^) revealed normal frequencies of naïve, decreased frequencies of effector and increased frequencies of memory T cells subsets in both CD4^+^ and CD8^+^ T cell fractions (Fig. 4G,H). Of note the reduction of effector and increase of memory subsets were more significant in the CD8^+^ T cells, when compared with CD4^+^ T cells (Fig. 4H). Finally, analysis of peripheral blood indicated a reduction of total CD3ε^+^ T cells in A20^Hem-KO^ mice (Fig. 4I). Together, these data indicated that T cell development in the thymus and maintenance in the periphery are affected in the absence of A20.

### Loss of A20 leads to altered differentiation of Lineage committed progenitors

**Figure 5:**
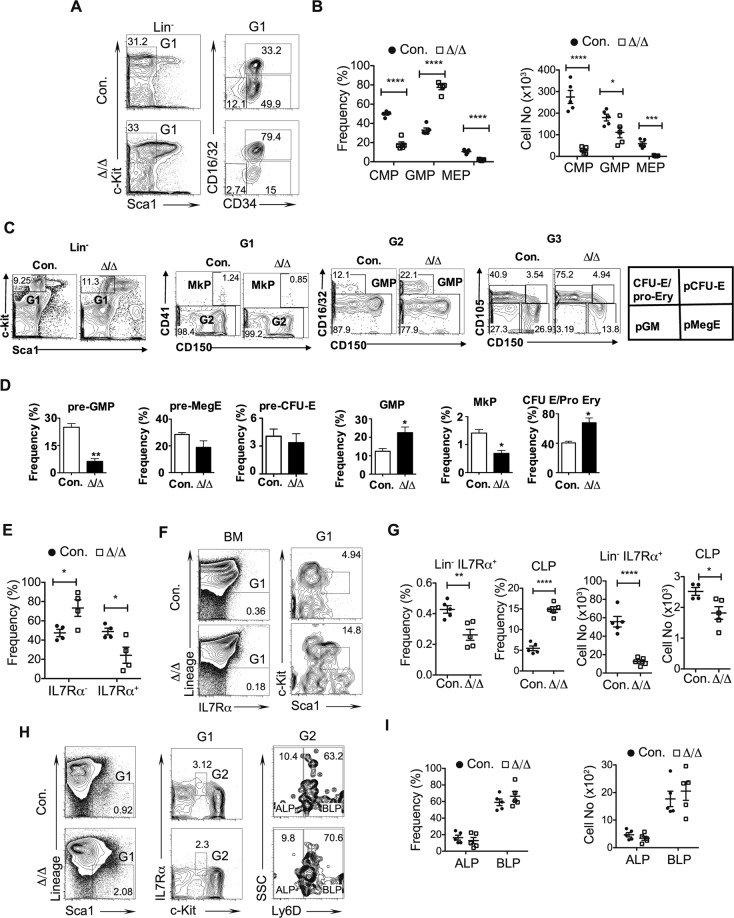
Disturbed differentiation of lineage committed progenitors in A20 deficient hematopoietic cells. (**A**,**B**) FACS plots (**A**), frequencies and absolute numbers (**B**) of CMPs, GMPs and MEPs from the BM of 14 days old A20^Hem-KO^ and control mice (n = 5). (**C**,**D**) FACS plots (**C**) and frequencies (**D**) of pre-GMP, pre-MegE, pre-CFU-E, GMP, MkP and CFU-E/pro-Ery from the BM of 14 days old A20^Hem-KO^ and control mice (n = 3). (**E**) Frequencies of IL7Rα^+^ LMPPs and IL7Rα^–^ LMPPs from the BM of 14 days old A20^Hem-KO^ and control mice (n = 4). (**F**,**G**) FACS plots (**F**), frequencies and absolute numbers (**G**) of Lin^−^IL7Rα^+^ cells and CLPs from the BM of 14 days old A20^Hem-KO^ and control mice (n = 5). (**H**,**I**) FACS plots (**H**), frequencies and absolute numbers (**I**) of ALPs and BLPs from the BM of 14 days old A20^Hem-KO^ and control mice (n = 5). All data represent mean ± SEM. Two-tailed student’s t tests were used to assess statistical significance (*P < 0.05, **P < 0.01, ***P < 0.001).

To investigate if the hematopoietic differentiation defects of A20^Hem-KO^ mice are associated with defective generation of lineage committed progenitors, we focused on the progenitors of myeloid, erythroid and lymphoid lineages. Flow cytometry studies on the previously reported^30^ Common Myeloid Progenitors (CMPs; Lin^−^Sca1^−^c-Kit^+^CD16/32^−^CD34^+^), Granulocyte-Monocyte Progenitors (GMPs; Lin^−^Sca1^−^c-Kit^+^CD16/32^+^CD34^+^) and Megakaryocyte Erythrocyte Progenitors (MEPs; Lin^−^Sca1^−^c-Kit^+^CD16/32^−^CD34^−^) indicated a decrease in the proportion of CMPs and MEPs, and an increased proportion of GMPs in A20^Hem-KO^ mice (Fig. 5A,B). Enumeration of absolute cell counts of these progenitors revealed a severe reduction of CMPs and MEPs, and a modest decrease of GMPs in the BM of A20^Hem-KO^ mice (Fig. 5B). To validate these findings through a more refined myeloid progenitor analyses, we followed the immunophenotype scheme established by Pronk *et al*.^31^. Accordingly, we noticed a reduction of pre-GMP, normal frequencies of pre-megakaryocyte-Erythrocyte (MegE) & pre-colony forming unit (CFU)-E, increased frequencies of GMP and CFU-E/pro-Ery, and reduced frequencies of megakaryocyte progenitors (MkPs) (Fig. 5C,D) in A20 deficient mice. Next, we quantified the frequencies of Lymphoid Primed Multipotent Progenitors (LMPPs; Lin^-^Sca1^+^c-Kit^+^Flt3^+^). Analysis of LMPPs revealed a reduction in the frequencies of cells that expressed IL7Rα in A20^Hem-KO^ mice (Fig. 5E). Consistently a reduction in, both relative and absolute numbers of, Lin^−^IL7Rα^+^ cells was noticed in the BM of A20^Hem-KO^ mice (Fig. 5F,G). Further studies on Common Lymphoid Progenitors (CLP; Lin^−^IL7Rα^+^Sca1^int^c-Kit^int^), a bona-fide precursor of the lymphoid lineage, indicated an increase in relative numbers, but a decrease in absolute numbers in the BM of A20^Hem-KO^ mice (Fig. 5F,G). Finally, we determined the frequencies of All Lymphoid Progenitors (ALP; Lin^-^Sca1^+^c-Kit^int^IL7Rα^+^Ly6D^−^) and B Lymphoid Progenitors (BLP; Lin^−^Sca1^+^c-Kit^int^IL7Rα^+^Ly6D^+^). Interestingly, these data indicated normal relative and absolute numbers of ALPs and BLPs in the BM of A20^Hem-KO^ mice (Fig. 5H,I). These data suggest that A20 deficiency affects early hematopoietic cell fate decisions, especially at the differentiation stage of lineage committed progenitors.

### A20 deficient myeloproliferation is cell extrinsic

**Figure 6:**
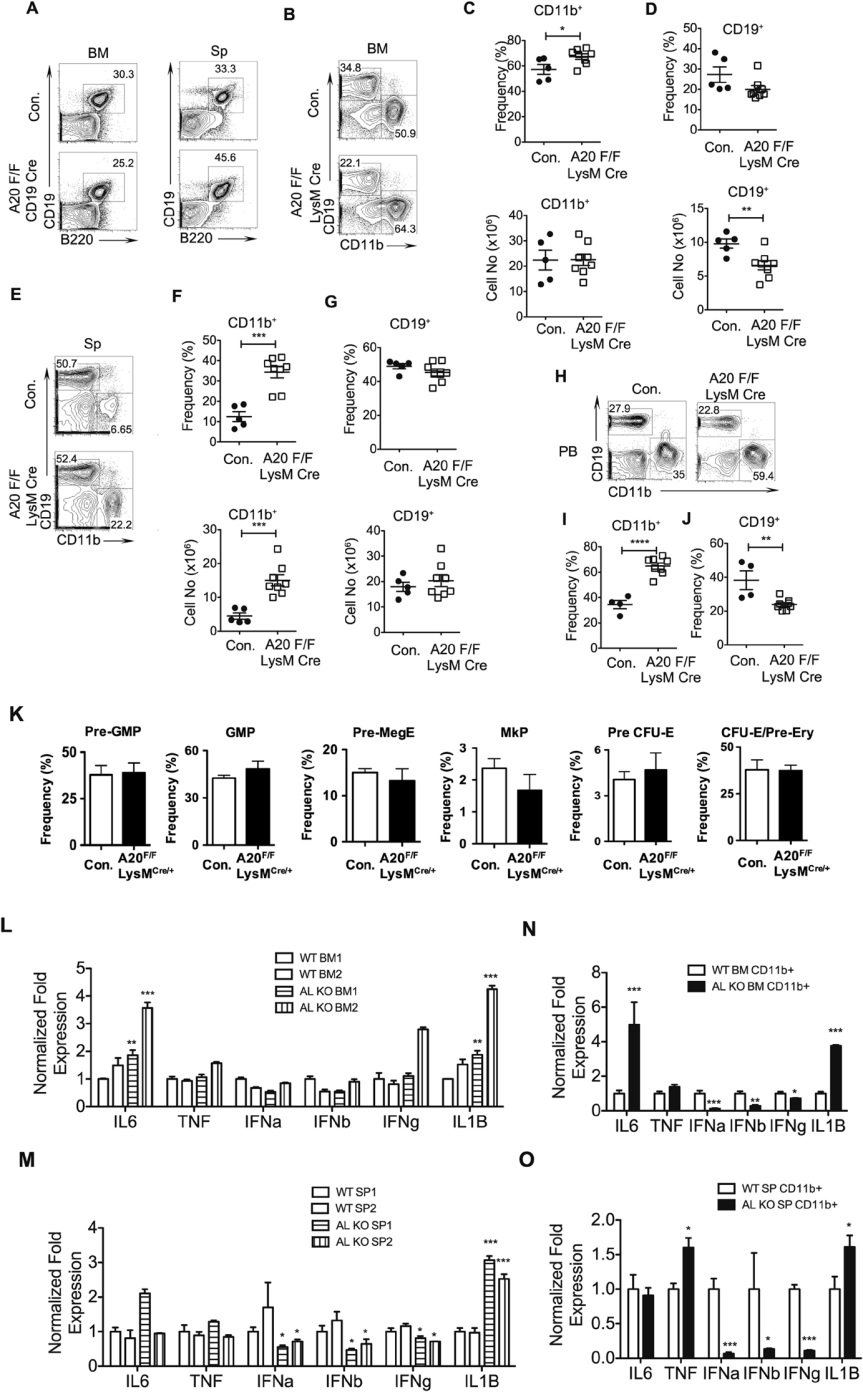
A20 deficiency in myeloid cells causes myeloproliferation. (**A**) FACS plots of B cells from the bone marrow (BM) and spleen (Sp) of 6 weeks old A20^F/F^CD19^cre/+^ and control mice. (**B**–**D**) FACS plots (**B**), frequencies and absolute numbers (**C**,**D**) of myeloid cells (**B**,**C**) and B cells (**B**,**D**) from the BM of 6 weeks old A20^F/F^LysM^cre/+^ and control mice (n = 5–8). (**E**–**G**) FACS plots (**E**), frequencies and absolute numbers (**F**,**G**) of myeloid cells (**E**,**F**) and B cells (**E**,**G**) from the spleen of 6 weeks old A20^F/F^LysM^cre/+^ and control mice (n = 5–8). (**H**–**J**) FACS plots (**H**), frequencies (**I**,**J**) of myeloid cells (**H**,**I**) and B cells (**H**,**J**) from the peripheral blood (PB) of 6 weeks old A20^F/F^LysM^cre/+^ and control mice (n = 4–8). (**K**) Frequencies of pre-GMP, pre-MegE, pre-CFU-E, GMP, MkP and CFU-E/pro-Ery from the BM of A20^F/F^LysM^cre/+^ and control mice (n = 5). (**L**,**M**) Real time PCR data of total cells from the BM (**L**) and spleen (**M**) of 6 weeks old A20^F/F^LysM^cre/+^ and control mice. Expression levels of target genes were normalized to HPRT levels. AL refers to A20^F/F^LysM^cre/+^ mice; BM1 & BM2 and SP1 and SP2 refer to two independent biological samples. (**N**,**O**) Real time PCR data for cytokines of myeloid cells from the BM (**N**) and spleen (**O**) of 6 weeks old A20^F/F^LysM^cre/+^ and control mice (n = 2–5). Expression levels of target genes were normalized to HPRT levels. All data represent mean ± SEM. Two-tailed student’s t tests were used to assess statistical significance (*P < 0.05, **P < 0.01, ***P < 0.001).

In order to understand the mechanisms that contribute to myeloproliferation and B cell defects in A20^Hem-KO^ mice, we crossed A20^floxed^ mice with either CD19^cre^ or LysM^cre^ transgenic mice, to specifically ablate A20 in B cells and myeloid lineage cells, respectively. Surprisingly, our analysis indicated normal frequencies of CD19^+^B220^+^ B cells (Fig. 6A), and no difference in CD11b^+^ myeloid cells (data not shown), in the BM and spleen of A20^F/F^CD19^cre/+^ mice (Fig. 6A). These data suggested that the B cell and myeloid differentiation defects observed in A20^Hem-KO^ mice are not caused due to lack of A20 in CD19^+^ cells. Next, we analyzed myeloid differentiation in A20^F/F^LysM^cre/+^ mice. Analysis of BM from these mice indicated a modest relative increase of CD11b^+^ myeloid cells (Fig. 6B,C), even though the absolute numbers of CD11b^+^ cells were normal in the BM of A20^F/F^LysM^cre/+^ mice (Fig. 6B,C). On the other hand, the relative frequencies of CD19^+^ cells were normal and the absolute numbers of CD19^+^ cells were decreased in the BM of A20^F/F^LysM^cre/+^ mice (Fig. 6D). Determination of frequencies of CD11b^+^ cells and CD19^+^ cells of the spleen documented a myeloproliferative phenotype with a remarkable increase, in both relative and absolute numbers, of CD11b^+^ cells in A20^F/F^LysM^cre/+^ mice (Fig. 6E,F). Nevertheless, the relative and absolute frequencies of CD19^+^ cells in the spleen of A20^F/F^LysM^cre/+^ mice were not altered (Fig. 6E,G). Analysis of peripheral blood from A20^F/F^LysM^cre/+^ mice indicated a significant increase of CD11b^+^ myeloid cells (Fig. 6H,I) and decrease of CD19^+^ B cells (Fig. 6H,J). Furthermore, detailed analysis of myeloid progenitors^31^ in the BM revealed no significant differences in the progenitors of myeloid/erythroid/megakaryocytic lineage of A20^F/F^LysM^cre/+^ mice (Fig. 6K).

Previous reports, including our own, demonstrated that A20 deficiency leads to decontrolled expression of inflammatory cytokines, due to exaggerated levels of NF-κB signals^11^. Based on the observation that B cell numbers are not altered in A20^F/F^CD19^cre/+^ mice but modestly altered in A20^F/F^LysM^cre/+^ mice, we hypothesized that the hematopoietic defects can be caused, at least in parts, by deregulated expression of inflammatory cytokines. Thus, we quantified the expression levels of pro-inflammatory cytokines in A20^F/F^LysM^cre/+^ mice. Real-Time PCR analysis of total cells of BM indicated increased expression of IL1β and IL6, normal expression of TNFα, IFNα, IFNβ and IFNγ (Fig. 6L). Analysis of spleen indicated elevated expression of IL1β and normal expression of IL6 and TNFα, and a modest reduction of IFNα, IFNβ and IFNγ (Fig. 6M) in A20^F/F^LysM^cre/+^ mice. Next, we analyzed the expression levels of pro-inflammatory cytokines in CD11b^+^ cells of the BM and Spleen, as this was the major cell type that was deficient for A20 in A20^F/F^LysM^cre/+^ mice. Real-Time PCR studies on purified CD11b^+^ cells of the BM revealed an augmented expression of IL6 and IL1β, normal levels of TNFα, and reduced expression of IFNα, IFNβ and IFNγ in A20^F/F^LysM^cre/+^ mice (Fig. 6N). On the other hand, analysis of purified CD11b^+^ cells of the spleen indicated elevated expression of TNFα and IL1β, normal levels of IL6 and reduced expression of IFNα, IFNβ and IFNγ in A20^F/F^LysM^cre/+^ mice (Fig. 6O). Collectively, these data suggest that A20 deficiency in the myeloid lineage causes myeloproliferation and suppress B cell differentiation, and that lack of A20 in CD19^+^ cells is not sufficient to suppress B cell differentiation in the BM.

### A20 deficient lymphopenia is caused by elevated IFNγ signals

**Figure 7:**
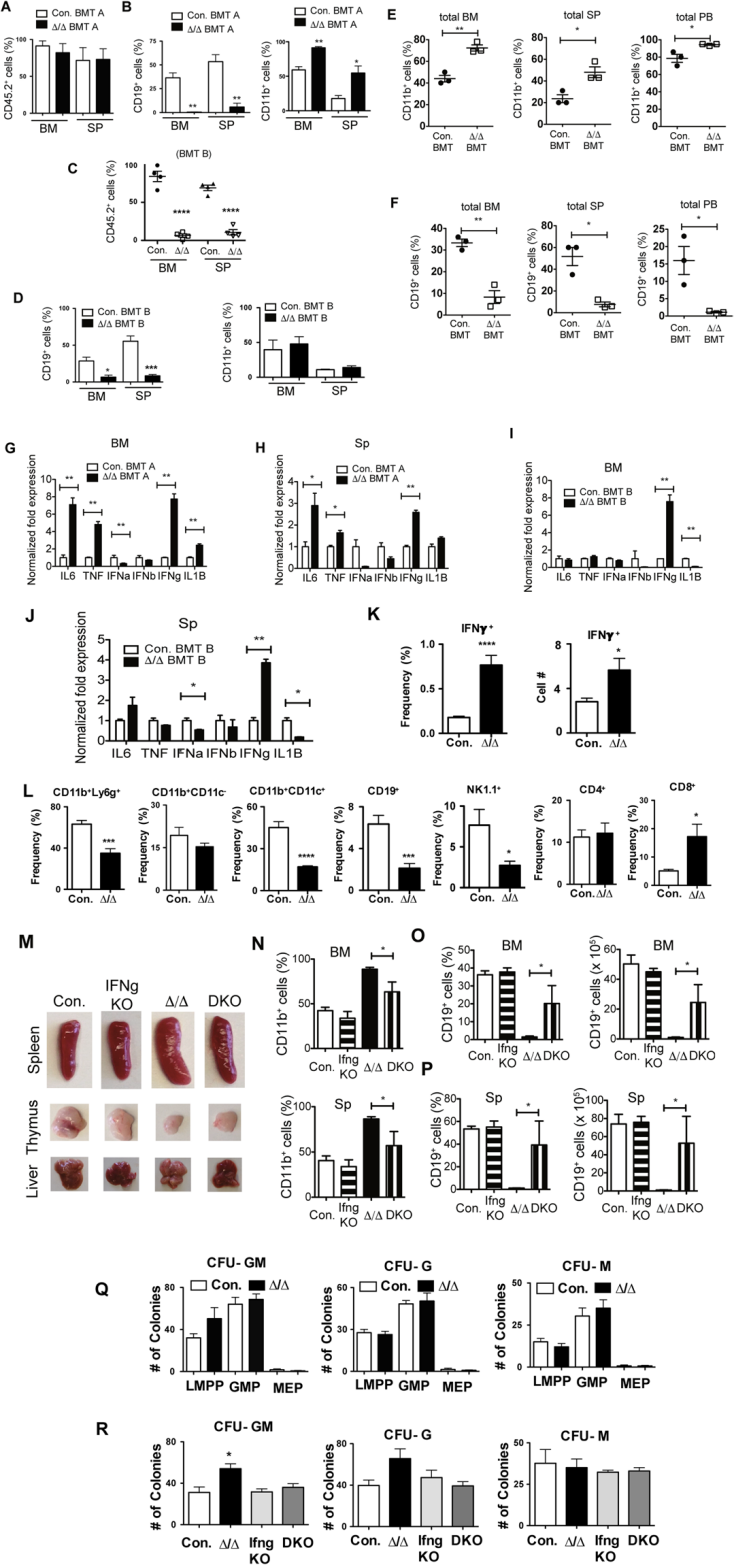
Loss of A20 causes lymphopenia via IFNγ signals. (**A**,**B**) Frequencies of donor (CD45.2^+^) derived hematopoiesis (**A**), and B cells & myeloid cells (**B**) in the bone marrow (BM) and spleen (SP) of congenic (CD45.1^+^) recipient mice (n = 3) that exhibit higher levels (>60%) of donor derived hematopoiesis (BMT-A). (**C**,**D**) Frequencies of donor (CD45.2^+^) derived hematopoiesis (**C**), and B cells & myeloid cells (**D**) in the BM and SP of congenic (CD45.1^+^) recipient mice (n = 4) that exhibit lower levels (<10%) of donor derived hematopoiesis (BMT-B). (**E**,**F**) Frequencies of myeloid cells (**E**) and B cells (**F**) in total BM (left panels), total spleen (middle panels) and total peripheral blood (right panels) from the recipients with mixed BM chimera containing Control:WT and A20^Hem-KO^:WT cells (n = 3). (**G**–**J**) Real time PCR data for cytokines of total cells from the BM (**G**,**I**) and spleen (**H**,**J**) of the mice with higher (**G**,**H**) and lower (**I**,**J**) donor-derived chimerism at 3 weeks after transplantation of either A20^Hem-KO^ or control mice. (**K**) Frequencies and absolute numbers of IFNγ^+^ splenocytes of A20^Hem-KO^ and control mice (n = 9). (**L**) Frequencies and absolute numbers of IFNγ^+^ immune subsets in the spleen of A20^Hem-KO^ and control mice (n = 9). (**M**) Pictures of Spleen, thymus and liver of control, IFNγ^−/−^ mice, A20^Hem-KO^ mice and DKO mice. (**N**) Frequencies of myeloid cells from the BM (upper panel) and spleen (lower panel) of control, IFNγ^−/−^ mice, A20^Hem-KO^ mice and DKO mice (n = 3). (**O**,**P**) Frequencies (left panels) and absolute numbers (right panels) of B cells from the BM (**O**) and spleen (**P**) of control, IFNγ^−/−^ mice, A20^Hem-KO^ mice and DKO mice (n = 3). Data represent two independent experiments. (**Q**) Colony Forming Unit (CFU) Assays of hematopoietic progenitor subsets from the BM of A20^Hem-KO^ mice and control mice. Data represent two independent experiments. (**R**) Colony Forming Unit (CFU) Assays of total BM from control, IFNγ^−/−^ mice, A20^Hem-KO^ mice and DKO mice. Data represent two independent experiments. All data represent mean ± SEM. Two-tailed student’s t tests were used to assess statistical significance (*P < 0.05, **P < 0.01, ***P < 0.001).

To identify if the hematopoietic defects of A20^Hem-KO^ mice are mediated by cell intrinsic or extrinsic factors, we performed bone marrow transplantation (BMT) experiments, as we reported earlier^11^. Even though recipients that received BM of A20^Hem-KO^ mice showed poor engraftment in most of the experiments, due to HSC defects^11^, there were a few A20^Hem-KO^ recipients in which the engraftment levels in the BM were ~90% and spleen were ~68%, at 3 weeks after transplantation (Fig. 7A). Analysis of total myeloid (CD11b^+^) cells and B (CD19^+^) cells (that include both donor (CD45.2^+^) and recipient (CD45.1^+^) derived) in these mice indicated a complete loss of B cell differentiation and increased myeloid differentiation in the BM (Fig. 7B) and a severe reduction of B cells and increased numbers of myeloid cells in the spleen (Fig. 7B) of A20^Hem-KO^ BM recipients. Of note, we were surprised by the lack of B cell differentiation in the BM of A20^Hem-KO^ recipients, because ~10% of cells in the BM and ~18% of cells in the spleen were recipient derived (CD45.1^+^) WT cells, which were capable of differentiating into B lineage cells.

Based on these findings, we hypothesized that A20^Hem-KO^ derived cells secrete some soluble factors (such as inflammatory cytokines) that are responsible for altered hematopoietic differentiation. To test this hypothesis, we analyzed hematopoietic differentiation in recipients that had very low levels of A20^Hem-KO^ derived hematopoiesis in the BM (~5%) and spleen (~20%) (Fig. 7C). Surprisingly, analysis of these mice indicated severe reduction of B cells in the BM (26% in control recipients and 1.9% in A20^Hem-KO^ recipients) and spleen (67% in control recipients and 11% in A20^Hem-KO^ recipients) (Fig. 7D). Furthermore, analysis of these mice indicated normal numbers of myeloid cells in both BM and spleen (Fig. 7D). These data suggested that the presence of ~5–20% A20^Hem-KO^ derived hematopoietic cells was sufficient to suppress B cell differentiation of 94% of WT (CD45.1^+^) cells in the BM. To further strengthen these findings, we generated recipients with mixed BM chimera containing Control:WT and A20^Hem-KO^:WT cells. Analysis of these mice revealed increased frequencies of myeloid cells in the BM, Spleen and Peripheral Blood (Fig. 7E), and severely reduced frequencies of B cells in the BM, Spleen and Peripheral Blood (Fig. 7F) of recipients that received A20^Hem-KO^ BM. Again, these data indicated that B cell differentiation capacity of WT BM cells was compromised in the presence of A20^Hem-KO^ BM cells.

Finally, we wished to identify the possible mechanisms by which A20 deficient hematopoietic cells suppress B cell differentiation pathways of WT HSPCs. For these studies, we analyzed the cells of our mixed chimera experiments (Fig. 7A,B). We analyzed the BM of WT recipients that exhibited better engraftment (~90% of A20^Hem-KO^ donor cells in the BM). Real-Time PCR studies on the expression levels of inflammatory cytokines indicated elevated expression levels of IL1β, IL6, TNFα and IFNγ, and reduced levels of IFNα in the BM of WT recipients that received A20^Hem-KO^ BM (Fig. 7G). Consistently, Real-Time PCR of spleens from these mice revealed increased expression levels of IL6, TNFα and IFNγ, and reduced expression of IFNα in recipients that received A20^Hem-KO^ BM (Fig. 7H). To corroborate these findings, we performed Real-Time PCR analysis of recipients that showed poor engraftment (~5% of A20^Hem-KO^ donor cells in the BM) (Fig. 7C,D). Interestingly, among the candidate inflammatory cytokines analyzed only IFNγ levels were elevated in the BM and spleen (Fig. 7I,J), whereas IL1β levels were reduced in the BM and spleen, of WT recipients that received A20^Hem-KO^ BM. To identify the immune cell type (s) that is responsible for the increased IFNγ levels in A20^Hem-KO^ mice, we performed flow cytometry based intracytoplasmic staining analyses. As expected, both the relative frequencies and the absolute numbers of IFNγ^+^ cells were augmented in the spleen of A20^Hem-KO^ mice (Fig. 7K). Further analyses, revealed that the frequencies of IFNγ^+^ granulocytes (CD11b^+^Ly6G^+^), dendritic (CD11b^+^CD11c^+^) cells, B (CD19^+^) cells and NK (CD19^+^) cells were reduced, myeloid/monocytes (CD11b^+^) and CD4^+^ T cells were normal and CD8^+^ T cells were remarkably increased in A20^Hem-KO^ mice (Fig. 7L). These findings, along with the results described in Fig. 7C,D provided a rationale for the hypothesis that the differentiation defects observed in A20 mice are caused due to exaggerated IFNγ signaling.

To validate this hypothesis, we genetically ablated IFNγ signals in A20^Hem-KO^ mice, by crossing A20^Hem-KO^ mice with IFNγ^−/−^ mice to generate A20^Hem-KO^IFNγ^−/−^ double KO (DKO) mice, as described earlier^11^. Interestingly, analysis of DKO mice indicated a rescue of splenomegaly, thymic atrophy and hepatomegaly phenotype (Fig. 7M) of A20^Hem-KO^ mice^11^. Analysis of BM and spleen indicated a significant reduction of CD11b^+^ myeloid cells, therefore a rescue of myeloproliferation phenotype of A20 deficient mice, in the DKO mice (Fig. 7N). Similarly, analysis for CD19^+^ B cells in the BM and spleen revealed normal frequencies and absolute numbers of B cells in the BM (Fig. 7O) and spleen (Fig. 7P) of DKO mice, therefore suggesting a rescue of B cell differentiation defects of A20^Hem-KO^ mice. To further strengthen these findings, we performed colony forming unit (CFU) assays using purified LMPPs, GMPs and MEPs from the BM of A20^Hem-KO^ mice. These studies documented that A20 deficient lineage restricted progenitors lack intrinsic capacities to form increased numbers of myeloid colonies in response to hematopoietic cytokines (Fig. 7Q). Finally, we performed CFU assays using total BM of A20^Hem-KO^ and DKO mice. These data demonstrated that ablating IFNγ expression in A20^Hem-KO^ mice suppresses the capacities to generate increased numbers of myeloid colonies from the BM of A20^Hem-KO^ mice (Fig. 7R). Taken together, these studies unequivocally demonstrated that decontrolled IFNγ signals are responsible, at least in part, for the onset of myeloproliferation and lymphopenia caused by A20 deficiency.

## Discussion

In the present study, we demonstrate that lack of A20 during hematopoiesis causes aberrant differentiation of lymphoid cells and increased differentiation of myeloid lineage cells. Interestingly, based on mixed BM chimera and depletion of A20 in lineage specific cells, our studies demonstrated that the differentiation defects are mainly caused by cell extrinsic mechanisms, especially due to exaggerated IFNγ signals. In keeping with previous findings^32–36^, our study identified IFNγ as a key factor responsible for the hematopoietic abnormalities of A20 deficient mice. We have previously shown that A20 deficiency leads to decontrolled expression of IFNγ, due to exaggerated binding of NF-κB to the regulatory sequences of IFNγ^11^. In the present study, we provide evidence on the physiological consequences of deregulated IFNγ signaling in hematopoietic differentiation pathways. Earlier studies indicated that inflammation affects the balance between myeloid versus lymphoid hematopoietic differentiation^37^. Interestingly, the frequencies of B lineage committed progenitors, including ALPs and BLPs, were similar between control and A20 deficient mice. Consistent with this observation, early stages of B cell development indicated normal frequencies of Fraction A and Fraction B, and modest reduction of Fraction C. However, a remarkable decrease was noticed at latter stages of B cell differentiation (Fraction E and Fraction F) and these defects are largely reversible in the absence of IFNγ. Based on these findings we hypothesize that in our model, IFNγ does not suppress lymphoid differentiation capacities of HSCs, instead it suppresses B cell differentiation by directly acting on B cell progenitors, especially at late stages of differentiation. Of note, a similar phenomenon was observed earlier in a murine model of IFNγ mediated suppression of B cells^34^. Even though the molecular mechanism responsible for this suppression has not been elucidated in our studies, based on the previous studies^32,38–40^, we speculate that IL7 signaling may be compromised due to increased IFNγ in A20 deficient mice. Indeed, transgenic expression of IFNγ results in reduced IL7 response in lymphocytes^32^. Recent studies demonstrated that IFNγ signaling results in elevated expression of SOCS1 during B cell development and it thereby inhibits IL7 responses^38,40^. Similarly, the striking reduction in overall cellularity of the thymus from A20 deficient mice^11^ can be explained by defective IL7 signaling caused by IFNγ. While mechanistic insights on the link between elevated IFNγ and perturbed thymopoiesis are missing, it is tempting to speculate that defective IL7 signaling caused by IFNγ (similar to the model established in B cells^38,40^) might be responsible for the severe thymic defects of A20 deficient mice.

During myeloid differentiation, IFNγ plays a critical role and affects developmental checkpoints between neutrophils and monocytes^40^. Mechanistic studies indicated that IFNγ induces expression of SOCS3, which in turn inhibit G-CSF induced activation STAT3, in GMPs that results in suppression of emergency granulopoiesis^39^ and activates expression of PU.1 and IRF-8 in GMPs to promote monocyte differentiation^41^. In addition to its direct role, IFNγ has also been shown to indirectly promote monocyte differentiation by inducing mesenchymal stromal cells to produce IL6^39^. IFNγ has also shown to inhibit erythrocyte differentiation by activating expression of PU.1, which physically interacts with Gata1 to suppress its functions in erythroid progenitors^39^. While the alteration of myeloid and erythroid differentiation in A20 deficient mice can be explained by elevated IFNγ signals, there are some findings that we believe are unique to A20 deficiency and are independent of IFNγ. These include; 1. A20 deficient mice show myeloproliferation, with increased numbers of both myelo/monocyte and granulocyte lineage cells; 2. Splenic erythropoiesis is augmented in A20 deficient mice; 3. Myeloid specific deletion of A20 results in myeloproliferation; and 4. Loss of both IFNγ and A20 shows a modest increase of myeloid cells in both bone marrow and spleen, although the myeloproliferation phenotype is rescued.

In essence, our study demonstrates that A20 plays an indispensable role in maintaining the myeloid vs. lymphoid differentiation potential of HSPCs, mainly by restricting decontrolled expression of pro-inflammatory cytokines. Our data also identified IFNγ as the key driver of A20 deficient myeloproliferation, anemia and lymphopenia. Even though we provide evidence that deregulated IFNγ signals are responsible for defective hematopoiesis, we believe that there are other distinct mechanisms, such as other direct targets of NF-κB, through which A20 may influence hematopoiesis. Future studies will be conducted to elucidate the molecular consequences that arise due to A20 deficiency during the development of immune system.

## Supplementary information

Supplementary information
