# Supplementary material for: A20 deficiency in hematopoietic stem cells causes lymphopenia and myeloproliferation due to elevated Interferon-γ signals
Source: Sci Rep. 2019 Sep 2;9:12658. doi: 10.1038/s41598-019-49038-8 (PMC6718430; doi:10.1038/s41598-019-49038-8)
Supplement: Supplementary file 1 — Supplementary information [file 41598_2019_49038_MOESM1_ESM.docx]

**A20 deficiency in hematopoietic stem cells causes lymphopenia and myeloproliferation due to elevated Interferon-**γ **signals**

**Masahiro Marshall Nakagawa and Chozha Vendan Rathinam**

| **Primer Name** | **Primer Sequence** |
| --- | --- |
| IL6_Forward | 5’ CTCTGCAAGAGACTTCCATCC 3’ |
| IL6_Reverse | 5’ TTCTGCAAGTGCATCATCGT 3’ |
| TNFa_Forward | 5’ TCTCAGCCTCTTCTCATTCCT 3’ |
| TNFa_Reverse | 5’ ACTTGGTGGTTTGCTACGAC 3’ |
| IFNa_Forward | 5’ SAWCYCTCCTAGACTCMTTCTGCA 3’ |
| IFNa_Reverse | 5’ TATDTCCTCACAGCCAGCAG 3’ |
| IFNb_Forward | 5’ GAATGGAAAGATCAACCTCACCT 3’ |
| IFNb_Reverse | 5’ ACAACAATAGTCTCATTCCACCC 3’ |
| IFNg_Forward | 5’ GAGCCAGATTATCTCTTTCTACCT 3’ |
| IFNg_Reverse | 5’ GTTGTTGACCTCAAACTTGGC 3’ |
| IL1b_Forward | 5’ TTTGACAGTGATGAGAATGACC 3’ |
| IL1b_Reverse | 5’ AATGAGTGATACTGCCTGCC 3’ |
| Hprt_Forward | 5’ AAGGACCTCTCGAAGTGTTGG 3’ |
| Hprt_Reverse | 5’ TTGCGCTCATCTTAGGCTTT 3’ |

**Supplementary Table 1: Primers used in the Realtime PCR studies.**
